# SOFA Score, Hemodynamics and Body Temperature Allow Early Discrimination between Porcine Peritonitis-Induced Sepsis and Peritonitis-Induced Septic Shock

**DOI:** 10.3390/jpm11030164

**Published:** 2021-02-28

**Authors:** Mahmoud Al-Obeidallah, Dagmar Jarkovská, Lenka Valešová, Jan Horák, Jan Jedlička, Lukáš Nalos, Jiří Chvojka, Jitka Švíglerová, Jitka Kuncová, Jan Beneš, Martin Matějovič, Milan Štengl

**Affiliations:** 1 Department of Physiology, Faculty of Medicine in Pilsen, Charles University, Alej Svobody 1655/76, 323 00 Pilsen, Czech Republic; alobeidallahm@fnplzen.cz (M.A.-O.); dagmar.jarkovska@lfp.cuni.cz (D.J.); jan.jedlicka@lfp.cuni.cz (J.J.); lukas.nalos@lfp.cuni.cz (L.N.); jitka.sviglerova@lfp.cuni.cz (J.Š.); jitka.kuncova@lfp.cuni.cz (J.K.); 2 Biomedical Center, Faculty of Medicine in Pilsen, Charles University, Alej Svobody 76, 323 00 Pilsen, Czech Republic; valesoval@fnplzen.cz (L.V.); horakjan@fnplzen.cz (J.H.); chvojkaj@fnplzen.cz (J.C.); benesj@fnplzen.cz (J.B.); matejovic@fnplzen.cz (M.M.); 3 Department of Internal Medicine I, Faculty of Medicine in Pilsen, Charles University, Alej Svobody 80, 304 60 Pilsen, Czech Republic; 4 Department of Aneshesiology and Intensive Care Medicine, Faculty of Medicine in Pilsen, Charles University, Alej Svobody 80, 304 60 Pilsen, Czech Republic

**Keywords:** sepsis, septic shock, SOFA score, pig

## Abstract

Porcine model of peritonitis-induced sepsis is a well-established clinically relevant model of human disease. Interindividual variability of the response often complicates the interpretation of findings. To better understand the biological basis of the disease variability, the progression of the disease was compared between animals with sepsis and septic shock. Peritonitis was induced by inoculation of autologous feces in fifteen anesthetized, mechanically ventilated and surgically instrumented pigs and continued for 24 h. Cardiovascular and biochemical parameters were collected at baseline (just before peritonitis induction), 12 h, 18 h and 24 h (end of the experiment) after induction of peritonitis. Analysis of multiple parameters revealed the earliest significant differences between sepsis and septic shock groups in the sequential organ failure assessment (SOFA) score, systemic vascular resistance, partial pressure of oxygen in mixed venous blood and body temperature. Other significant functional differences developed later in the course of the disease. The data indicate that SOFA score, hemodynamical parameters and body temperature discriminate early between sepsis and septic shock in a clinically relevant porcine model. Early pronounced alterations of these parameters may herald a progression of the disease toward irreversible septic shock.

## 1. Introduction

Sepsis is a major health problem worldwide, the incidence of sepsis continues to rise, and despite the various options of the spectrum of available interventions, the progression of sepsis to severe refractory septic shock remains a severe clinical condition with high mortality [1,2,3,4]. A meta-analysis of studies from developed high-income countries revealed global annual estimates of 31.5 million sepsis and 19.4 million severe sepsis cases, with potentially 5.3 million deaths in the hospital setting [5]. According to recent estimates, the rate of severe sepsis hospitalizations doubled during the last decade, resulting in more than 250,000 deaths in the United States yearly [6,7].

Early diagnosis of sepsis and a mechanistic understanding of the progression of septic disease, based on a monitoring of the functions of various organ systems, could allow effective early therapeutic interventions and prevent refractory septic shock [8,9,10]. However, despite intensive experimental and clinical efforts, the precise mechanisms of sepsis progression and transition into refractory septic shock remain unclear, limiting the optimal timing and success of therapeutic interventions.

According to the Third International Consensus Definitions for Sepsis and Septic Shock [8], sepsis is currently defined as a life-threatening organ dysfunction caused by a dysregulated host response to infection, whereas a septic shock represents a subset of sepsis in which particularly profound circulatory, cellular and metabolic abnormalities associated with a greater risk of mortality occur [8]. The organ dysfunction is quantified using the sequential (sepsis-related) organ failure assessment (SOFA) score, which is based on evaluation and scoring of several vital organ systems. Although the system reflects an up-to-date view of pathobiology and offers easily and objectively measurable clinical criteria, it has inherent limitations in the detection of early disease stages, in which organ failure is not fully developed yet. Furthermore, a more detailed analysis of organ function beyond the SOFA score criteria might provide valuable insights into the pathophysiological mechanisms of the disease. Finally, proper application of the consensus definitions (SOFA score criteria) to experimental animal research represents a significant challenge that will require translational analysis of interspecies differences in all relevant physiological and pathophysiological mechanisms [9].

Therefore, in this study, the progression of multiple organ dysfunction was carefully monitored in established, clinically relevant porcine models of peritonitis-induced sepsis and peritonitis-induced septic shock. Disease progression in these two groups was compared in search of critical early mechanisms responsible for the transition from sepsis to septic shock.

## 2. Materials and Methods

Animal handling and experiments complied with the European Directive for the Protection of Vertebrate Animals Used for Experimental and Other Scientific Purposes (86/609/EU) and were approved by the Committee for Experiments on Animals of the Charles University Faculty of Medicine in Pilsen. Fifteen farm pigs (Prestice Black-Pied pig) of either sex and of similar weight (40 ± 6 kg) were used for experiments. Sepsis (5 barrows, 3 sows) or septic shock (3 barrows, 4 sows) were induced by fecal peritonitis.

### 2.1. Anesthesia and Instrumentation

The protocols were described previously in detail [10]. In short, i.m. tiletamine (2.2 mg/kg), zolazepam (2.2 mg/kg) and xylazine (2.2 mg/kg) together with i.v. propofol 2% (1–4 mg/kg) and fentanyl (5–10 µg/kg/h) were used for anesthesia induction and maintenance. Mechanical ventilation (FiO_2_ 0.3, PEEP 8 cm H_2_O, tidal volume 10 mL/kg) was adjusted to maintain end/tidal pCO_2_ between 4 and 5 kPa. For muscle paralysis, i.v. rocuronium (4 mg for induction, 0.2–0.4 mg/kg/h for maintenance) was administered. A Ringerfundin solution (B. Braun Melsungen AG, Melsungen, Germany, 7 mL/kg/h) and 10% glucose infusion (1–4 mL/kg/h) were infused to maintain normovolemia and normoglycemia. The femoral artery, pulmonary artery and triple lumen central venous catheters were used for hemodynamic monitoring and blood sampling. Ultrasound flowprobe (Transonic Systems, Ithaca, NY) around the left renal artery was used for monitoring renal blood flow. Feces were inoculated through silicone drains into Morison and Douglas anatomical spaces.

### 2.2. Experimental Protocol

Experimental protocols were described previously [10,11]. In short, autologous feces (1 g/kg for inducing septic shock or 0.5 g/kg for inducing sepsis) were inoculated in the abdominal cavity after short cultivation (10 h, isotonic saline, 37 °C). The selection of doses was based on earlier studies of our group [11,12,13,14]. The high dose (1 g/kg) was sufficient in our experimental setting for the development of irreversible septic shock with sustained vasopressor support within 20–22 h. The low dose (0.5 g/kg) invariably did not induce septic shock within 24 h (no sustained vasopressor support, low plasma levels of lactate). Continuous i.v. norepinephrine was administered at mean arterial pressure (MAP) levels below 65 mmHg to return and maintain MAP above 70 mmHg. The total duration of the experiment was 34 h (4 h of instrumentation, 6 h of recovery and 24 h of peritonitis/sepsis/septic shock progression). The animals were carefully monitored by an experienced researcher throughout the experiment. The pigs were euthanized by anesthetic overdose and a subsequent necropsy with tissue sampling was performed.

### 2.3. Measurements

Hemodynamics and lead II ECG measurements were described previously [10,11,13]. The SOFA score, according to the SEPSIS-3 definitions [8], was modified by the exclusion of the Glasgow coma scale-based neurologic component with regard to general anesthesia. Datasets were recorded at baseline/sepsis induction (time point 1, TP1), 12 h (TP2), 18 h (TP3) and 24 h (TP4) after peritonitis induction. POCT analyses (Cobas B 123, Roche, Diagnostics, USA) of arterial blood were performed. Creatinine, liver enzymes and total protein serum levels were determined. ELISA methods were used for the determination of cytokines (Porcine Quantikine ELISA Kit, R&D System, Minneapolis, USA) and 8-isoprostane (Cayman Chemical, Michigan, USA). High-resolution respirometry (Oroboros Instruments, Innsbruck, Austria) was used for assessment of mitochondrial function by measuring oxygen consumption at 37 °C. Samples were obtained at the baseline by biopsy and, at the end of experiment, by organ dissection. Respiratory states and activities were determined as described elsewhere [15].

### 2.4. Statistical Analysis

Results are presented as means ± SD. The two-way mixed-design ANOVA followed by a post hoc Tukey test (OriginPro 2017, OriginLab Corp., Northampton, MA, USA) was used for comparing datasets. Values of *p* < 0.05 were considered significant.

## 3. Results

**Figure 1:**
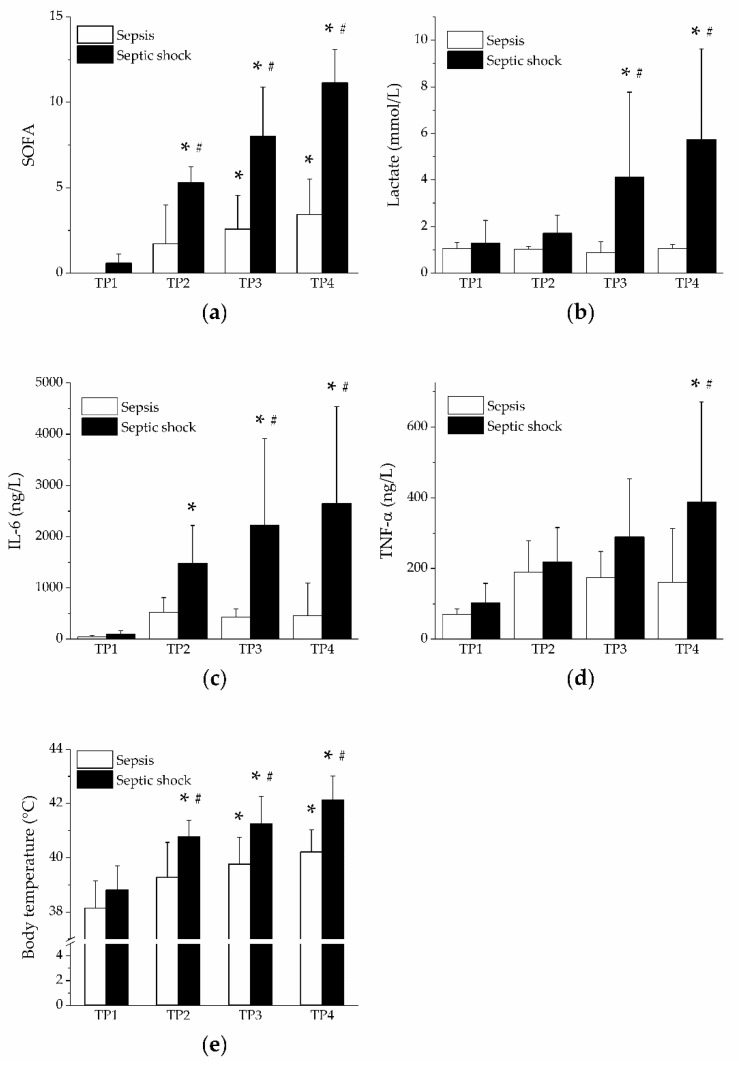
Sequential organ failure assessment (SOFA) score, lactate, cytokines and body temperature. Open columns, sepsis; filled columns, septic shock. *, *p* < 0.05 vs. baseline (TP1); #, *p* < 0.05 vs. sepsis. (**a**) SOFA score in sepsis and septic shock; (**b**) plasma levels of lactate in sepsis and septic shock; (**c**) plasma levels of IL-6 in sepsis and septic shock; (**d**) plasma levels of TNF-α in sepsis and septic shock; (**e**) body temperature in sepsis and septic shock.

Analysis of the SOFA score revealed faster progression of organ failure in the group of septic shock, as expected (Figure 1a). The rise of SOFA score values was found in the group of septic shock, followed by increased plasma levels of lactate (Figure 1b) and cytokines IL-6 (Figure 1c) and TNF-α (Figure 1d). The rise in body temperature was faster and more pronounced in the group of septic shock (Figure 1e).

**Figure 2:**
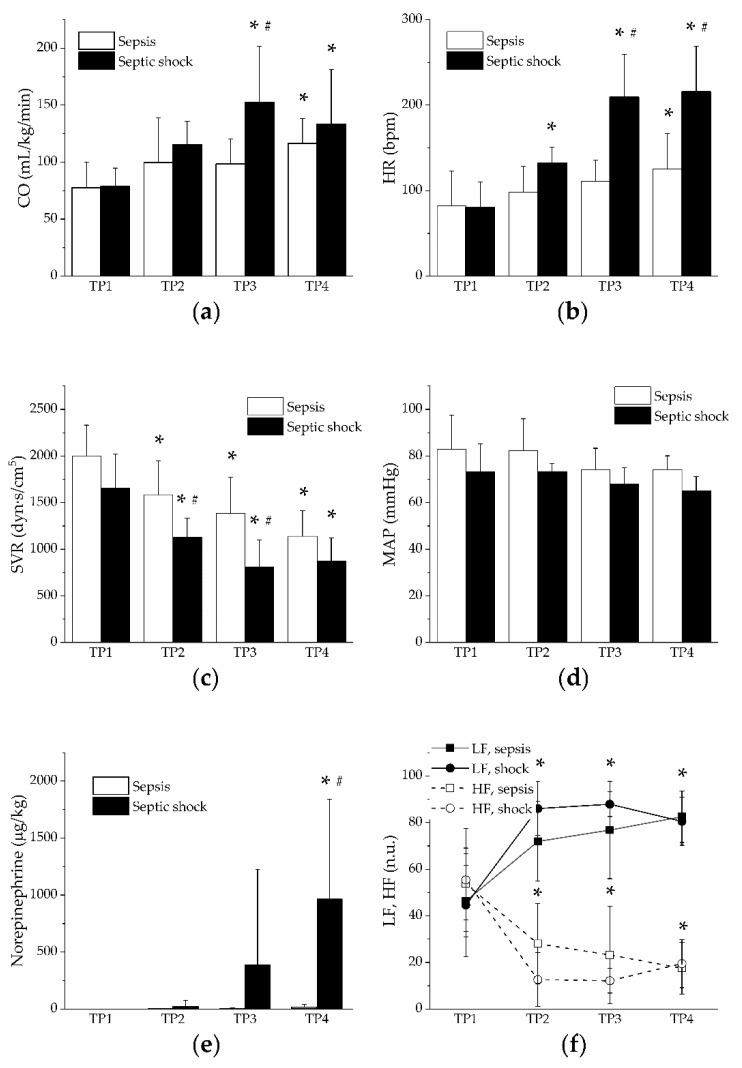
Hemodynamics and vasopressors. Open columns, sepsis; filled columns, septic shock. *, *p* < 0.05 vs. baseline (TP1); #, *p* < 0.05 vs. sepsis. (**a**) Cardiac output in sepsis and septic shock; (**b**) heart rate in sepsis and septic shock; (**c**) systemic vascular resistance in sepsis and septic shock; (**d**) mean arterial pressure in sepsis and septic shock; (**e**) norepinephrine dose in sepsis and septic shock. (**f**) low-frequency (LF) and high-frequency (HF) bands in sepsis and septic shock. Open symbols, HF. Filled symbols, LF.

Hyperdynamic circulation with high cardiac output (Figure 2a), tachycardia (Figure 2b) and peripheral systemic vasodilation (Figure 2c) developed in both groups, faster and to a higher extent in the group of septic shock. Mean arterial pressure remained stable throughout the entire experiment (Figure 2d), probably due to vasopressor support in the group of septic shock (Figure 2e) and a shift of autonomic nervous control toward sympathetic dominance in both groups as documented by the frequency domain analysis of heart rate variability (decreased high frequency band and increased low frequency band, Figure 2f). Central venous pressure and mean pulmonary artery pressure significantly increased only in the group of septic shock (Table 1). Pulmonary artery occlusion pressure and pulmonary vascular resistance were not influenced in either experimental group (Table 1).

**Figure 3:**
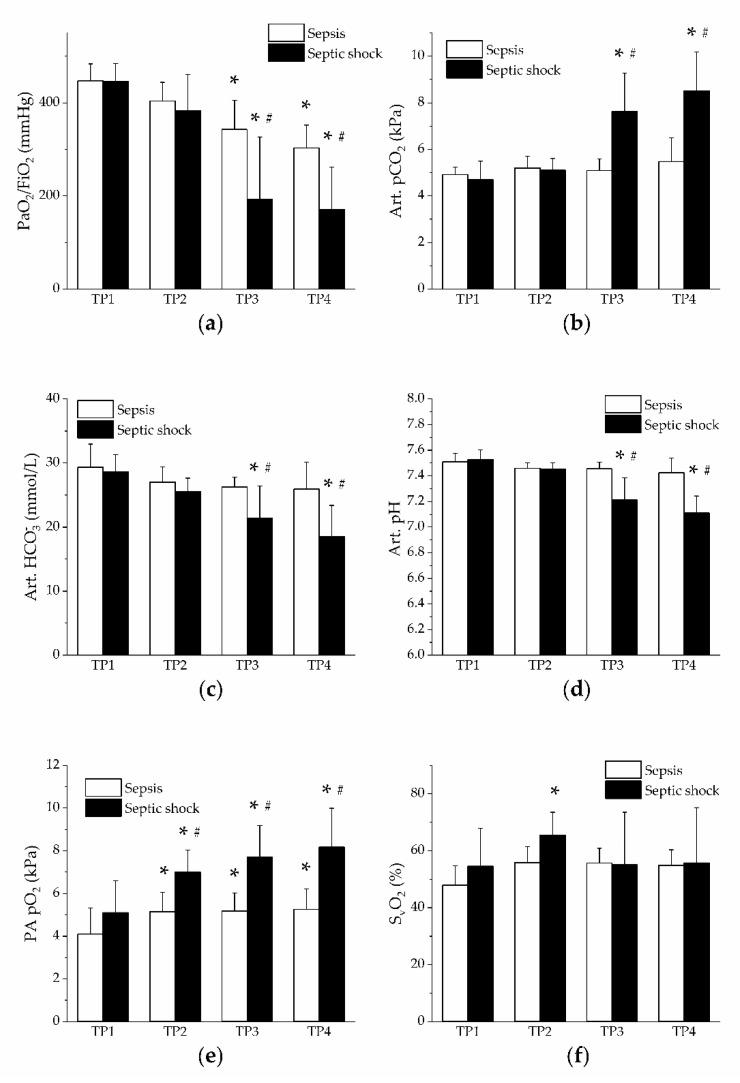
Respiratory gases and acid-base balance. Open columns, sepsis; filled columns, septic shock. *, *p* < 0.05 vs. baseline (TP1); #, *p* < 0.05 vs. sepsis. (**a**) PaO_2_/FiO_2_ ratio in sepsis and septic shock; (**b**) arterial pCO_2_ in sepsis and septic shock; (**c**) arterial bicarbonate in sepsis and septic shock; (**d**) arterial pH in sepsis and septic shock; (**e**) mixed venous (pulmonary artery, PA) pO_2_ in sepsis and septic shock; (**f**) mixed venous (pulmonary artery) O_2_ saturation (SvO_2_) in sepsis and septic shock.

The PaO_2_/FiO_2_ ratio was decreased in both groups, at time points 3 and 4, to a significantly higher extent in the group of septic shock (Figure 3a). In the group of septic shock, arterial pCO_2_ was increased, whereas arterial bicarbonate levels and pH were reduced (Figure 3b–d). Mixed venous blood pO_2_ showed an early increase in both groups, significantly more in the group of septic shock (Figure 3e), although the saturation was not significantly affected (Figure 3f). Oxygen delivery was increased in the group of septic shock (from the time point 3), whereas oxygen consumption was not affected in either group (Table 1).

**Figure 4:**
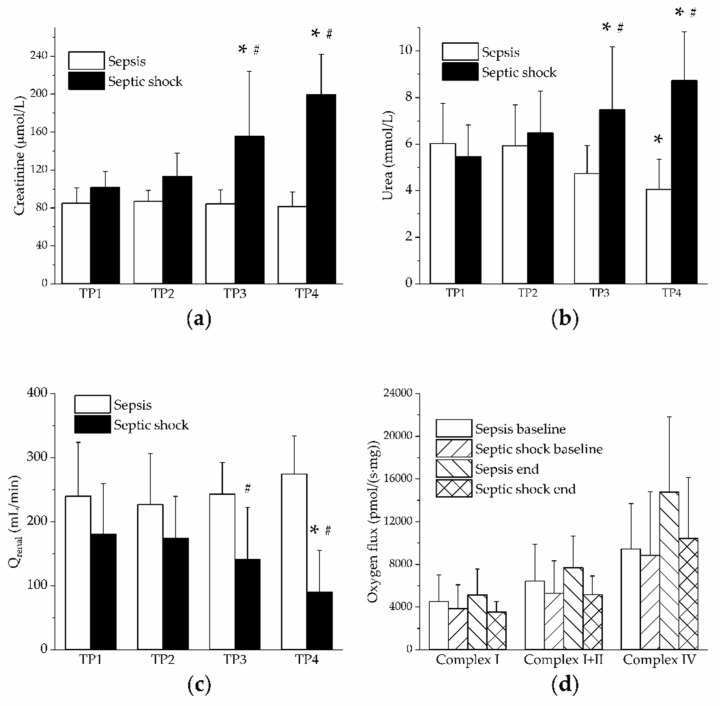
Renal functions. Open columns, sepsis; filled columns, septic shock. *, *p* < 0.05 vs. baseline (TP1); #, *p* < 0.05 vs. sepsis. (**a**) Creatinine plasma levels in sepsis and septic shock; (**b**) Urea plasma levels in sepsis and septic shock; (**c**) Renal blood flow in sepsis and septic shock; (**d**) Oxygen consumption by mitochondrial complexes I, I+II and IV measured by ultrasensitive oxygraphy. Group of sepsis at baseline and at the end of the experiment. Group of septic shock at baseline and at the end of the experiment.

In the group of septic shock, increased plasma levels of creatinine and urea together with reduced renal blood flow were found (Figure 4a–c). Nevertheless, renal mitochondrial respiration (activities of Complex I, II and IV) assessed by high-resolution respirometry was not affected in either group (Figure 4d). Urine output was maintained stable throughout the experiment in both groups (Table 1).

Increased plasma levels of ALP and AST suggest hepatic injury in the group of septic shock (Table 1). Platelet counts were reduced in both groups to a similar extent (Table 1). No significant changes in plasma levels of isoprostanes or TBARS were found in either group (Table 1). Nitrogen oxides (NOx) plasma levels gradually increased in both groups (Table 1).

## 4. Discussion

Analysis of SOFA score revealed significant differences between the groups of sepsis and septic shock already at the time point 2 (12 h after induction of peritonitis). In search of relevant mechanisms responsible for the early differential progression of the disease, a number of organ functions were analyzed, and significant early differences were only found in the cardiovascular system and in body temperature. In the cardiovascular system, systemic vascular resistance was decreased and mixed venous (pulmonary artery) blood pO_2_ increased, both changes being more pronounced in the group of septic shock. Reduction of the systemic vascular resistance preceded significantly the therapeutic administration of vasopressor (norepinephrine), which is (together with mean arterial pressure) the only criterion of cardiovascular dysfunction in the SOFA score. Perhaps the inclusion of the systemic vascular resistance, a surrogate marker of sepsis-induced vascular dysfunction, to the cardiovascular assessment algorithm, could enhance the sensitivity of the SOFA score, especially in the early phases of the disease.

In general, the higher levels of mixed venous pO_2_ could reflect a shift of the oxygen dissociation curve and/or a lower O_2_ extraction by the tissues. The available evidence for lower O_2_ extraction in sepsis, which can be due to increased microcirculatory shunting or mitochondrial dysfunction [16], is rather abundant. For shunting, a markedly decreased functional microvessel density and mean erythrocyte velocity were reported in the gut and sublingual capillary beds of septic pigs [17], and decreased microvascular oxygen partial pressures of ileal serosa and mucosa were found in endotoxemic pigs [18], and pathological flow distribution in skeletal muscle microcirculation was found in rat cecal ligation and puncture model of sepsis [19]. Mitochondrial dysfunction in sepsis was demonstrated in various organs and tissues [11,20,21]. However, the precise role of mitochondria in particular sepsis-induced organ dysfunction still remains unclear [12].

In our study, however, higher levels of mixed venous pO_2_ were not accompanied by corresponding changes in oxygen saturation and oxygen extraction ratio. Furthermore, similar levels of renal mitochondrial respiration were found in both sepsis and septic shock. All these findings argue against the significant contribution of microvascular shunting and mitochondrial dysfunction and, rather, indicate a shift of the oxygen dissociation curve as the most likely mechanism of higher mixed venous pO_2_. Since pCO_2_ levels in mixed venous blood were at the time point 2 similar in sepsis and septic shock, the shift of the oxygen dissociation curve was probably induced by elevated body temperature, which developed earlier and was more pronounced in the group of septic shock. In a novel scoring system for predicting in-hospital mortality of sepsis patients based on a prospective, observational multicenter study, fever was identified as one of the predictors with a strong correlation [22]. Fever had positive effects on sepsis patients, probably due to antimicrobial effects and stimulation of innate immunity [23,24,25].

The underlying mechanisms of decreased systemic vascular resistance in sepsis remain unclear. In general, the vascular smooth muscle tone is a product of intensive crosstalk of local (humoral) regulatory mechanisms and of central regulatory mechanisms of the autonomic nervous system, dominantly the sympathetic branch. Analysis of heart rate variability, in agreement with our previous studies [14,26], revealed a shift of the sympathovagal balance toward sympathetic dominance (increase in LF component, decrease in HF component) in both groups to a similar extent. However, the increased sympathetic drive, in the group of septic shock even with further support of therapeutic administration of norepinephrine, did not override (peripheral) vasodilatory mechanism, and consequently, it did not prevent a marked reduction of the systemic vascular resistance. The sepsis-induced increase in plasma levels of nitrogen oxides was rather slow and similar in both groups, thus arguing against the dominant role of the nitric oxide-related pathway in early vasodilation. However, local heterogeneity in the production of nitric oxide that might contribute to pathological flow distribution cannot be excluded.

Direct vasodilatory effects of inflammatory mediators and other signaling molecules should also be taken into account. The central player of inflammatory pathways, transcription factor NF-kB, can be stimulated by a number of inflammatory cytokines, including TNF-α [27]. Activation of NF-kB in vascular smooth muscle cells was shown to potently downregulate smooth muscle cell contractile genes [28], which might contribute to vasodilation. However, from the vast number of possible NF-kB activators, only plasma levels of TNF-α were determined, and with regard to the slow dynamics of TNF-α rise, a significant contribution of this pathway does not seem likely. For many vasoactive substances, both vasodilatory and vasoconstrictory, increased plasma levels in sepsis were reported. Lysozyme-c, a product of the disintegration of leukocytes from the spleen and other organs, was reported to contribute to both myocardial depression and arterial vasodilation in the canine model of septic shock through H_2_O_2_ signaling [29]. In addition, plasma levels of adrenomedullin, a free circulating peptide involved in the regulation of endothelial barrier function and vascular tone, were found to be increased in sepsis and to correlate with the extent of vasodilation as well as with disease severity and mortality [30]. Increased plasma levels of a vasoactive intestinal peptide that causes profound and long-lasting relaxation of the vascular smooth muscle were described in porcine peritonitis-induced sepsis [31]. In a series of studies, reduced responsiveness of the resistance arterioles of septic rats to several vasoconstrictors (vasopressin, norepinephrine, endothelin-1) was demonstrated [32,33,34]. Taken together, dynamic interactions of many vasoactive agents are probably involved and may result in pathological vasodilation in various tissues and organs.

Hypoxia per se may also induce vasodilation to allow proper tissue metabolic coupling between oxygen supply and demand. The phenomenon was well described in skeletal muscle, and it is mediated through several mechanisms with a central role of adenosine [35]. During hypoxia, adenosine is released from the endothelium and, through endothelial receptors and opening of ATP-sensitive K^+^ channels, it produces vasodilation in a nitric oxide-dependent manner. Adenosine also attenuates the vasoconstrictor effects of a sympathetic system and exerts a number of immunomodulatory and cytoprotective effects. In sepsis, however, the cardiovascular effects of adenosine are variable, according to the receptor activated, the vascular bed and the time elapsed [36]. Regardless, protective effects of adenosine A1 and A3 receptor subtypes activation with reduced mortality, improved renal and hepatic function and reduced inflammation were demonstrated in a mouse model of cecal ligation and puncture-induced sepsis [37].

Using the SOFA score [8], animals with septic shock could be well distinguished from those with sepsis, indicating a good translatability of human sepsis criteria to the porcine model and confirming the clinical relevance of the model. Contribution of the cardiovascular system to the SOFA classification, however, seems underrepresented. Only mean arterial pressure and the need for vasopressor support are currently involved in the cardiovascular branch of the classification. The inclusion of additional cardiovascular parameters like systemic vascular resistance might perhaps increase the early sensitivity and predictive power of the classification.

### Study Limitations

Mitochondrial respiration was only measured in the kidney at the end of in vivo experiment (24 h after peritonitis induction). Although no significant differences between groups of sepsis and septic shock were found in the kidney, we cannot exclude differential mitochondrial remodeling at earlier time points and/or in other tissues [38].

The SOFA score was determined according to human sepsis criteria [8]. Slight physiological interspecies differences might influence the relative value of individual parameters in a porcine model. Furthermore, with regard to the general anesthesia used throughout the experiment, the neurological Glasgow Coma Scale score was omitted, which might underestimate the contribution of the central nervous system. A hyperdynamic phenotype of the porcine peritonitis-induced sepsis model limits application of the results to the hypodynamic phenotypes.

The quick SOFA score (qSOFA) allows simple identification of adult patients with suspected infection and probably poor outcome based on three bedside criteria (altered mentation, hypotension and tachypnea). Since similar predictive validity of qSOfa and SOFA scores was demonstrated in patients [39], it would be worthwhile to compare these scores in our porcine model. Unfortunately, in our experimental setting, the qSOFA score could not be determined since the animals are anesthetized and mechanically ventilated throughout the experiment, which precludes any meaningful analysis of two (out of three) qSOFA criteria (mentation and tachypnea).

Oxidative stress contributes to the pathophysiology of sepsis [40]. However, in the porcine model of peritonitis-induced sepsis, the systemic plasma levels of oxidative stress markers TBARS and isoprostanes were not increased, which might limit the clinical relevance of the model. Local tissue elevations of the markers (and of oxidative stress), however, cannot be excluded.

In this study, the organ systems included in the SOFA score were dominantly analyzed. Contributions of other organs and tissues were neglected, although they might, in particular settings, also be of importance (e.g., gastrointestinal tract, [41]).

A longer duration of experiment could reveal a delayed progression into the septic shock also in the group of sepsis, and a detailed comparison of differential disease dynamics would certainly be of clinical interest. However, with regard to the extensive requirements of anesthesia, instrumentation and bedside care during the experiment, a 24 h period was chosen as the optimal compromise of experimental and clinical relevance and of technical and human-resource demands.

## 5. Conclusions

In a clinically relevant porcine model of peritonitis-induced sepsis and septic shock, comparisons of disease progression between animals with sepsis and animals with septic shock revealed early significant differences in the SOFA score, systemic vascular resistance, body temperature and the partial pressure of oxygen in mixed venous blood. Early pronounced alterations of these parameters may herald a progression of the disease toward irreversible septic shock.

## Figures and Tables

**Table 1:** Hemodynamical and biochemical parameters in sepsis and septic shock. SV: stroke volume, PAOP: pulmonary artery occlusion pressure, CVP: central venous pressure, MPAP: mean pulmonary artery pressure, DO_2_: oxygen delivery, VO_2_: oxygen consumption, O_2_ER: oxygen extraction ratio, ALP: alkaline phosphatase, AST: aspartate transaminase, ALT: alanine transaminase, TBARs: thiobarbituric acid reactive substances. NOx: nitrogen oxides. Mean ± SD; *, *p* < 0.05 vs. baseline (TP1); #, *p* < 0.05 vs. sepsis.

Parameter	Sepsis				Septic Shock			
	TP1	TP2	TP3	TP4	TP1	TP2	TP3	TP4
SV (mL)	40 ± 12	39 ± 6	35 ± 6	39 ± 12	44 ± 19	35 ± 7	33 ± 15	29 ± 17
PAOP (mmHg)	10 ± 3	9 ± 2	11±2	13±3	9 ± 2	10 ± 1	12 ± 3	11 ± 6
CVP (mmHg)	10 ± 2	10 ± 3	11 ± 3	12 ± 4	10 ± 3	11 ± 2	15 ± 2 *,#	14 ± 3 *
MPAP (mmHg)	22 ± 5	24 ± 3	24 ± 3	26 ± 6	23 ± 3	24 ± 3	33 ± 11 *,#	34 ± 11 *,#
DO_2_ (mL/(min·kg))	9.5 ± 3.6	12.3 ± 4.3	10.5 ± 3.7	11.8 ± 3.5	9.1±1.1	15.2 ± 2.3	21.5 ± 7.1 *,#	18.6 ± 6.4 *
VO_2_ (mL/(min·kg))	4.7 ± 1.4	4.2 ± 1.6	4.6±1.3	4.7 ± 0.9	4.1 ± 1.2	5.2 ± 1.5	6.9 ± 1.5	6.1 ± 1.3
O_2_ER	0.51 ± 0.07	0.35 ± 0.12	0.44±0.05	0.41 ± 0.06	0.45±0.13	0.34 ± 0.08	0.35 ± 0.13	0.33 ± 0.17
Fluid resuscitation (mL)	1883 ± 273	3999 ± 1058	3042 ± 491	2554 ± 498	1889 ± 466	4223 ± 738	2488 ± 916	1628 ± 1325 #
Hemoglobin (g/L)	77 ± 31	81 ± 33	56 ± 40	66 ± 28	59 ± 33	72 ± 43	83 ± 50	44 ± 52 *,#
Urine output (mL)	267 ± 85	409 ± 248	468 ± 346	537 ± 315	248 ± 59	303 ± 114	332 ± 243	314 ± 371
ALP (μkat/L)	1.9 ± 0.2	1.9 ± 0.4	1.7 ± 0.3	1.3 ± 0.3	1.9 ± 0.4	2.1 ± 0.5	3.1 ± 1.6	3.4±1.5 *,#
AST (μkat/L)	1.3 ± 0.5	1.4 ± 0.4	1.5 ± 0.4	1.5 ± 0.6	0.7 ± 0.1	1.8 ± 0.8	3.0 ± 2.2 *	3.3±2.0 *
ALT (μkat/L)	0.7 ± 0.1	0.5 ± 0.1	0.6 ± 0.1	0.6 ± 0.1	0.6 ± 0.1	0.5 ± 0.1	0.6 ± 0.2	0.7 ± 0.32
Bilirubin (µmol/L)	3 ± 0	3 ± 0	3 ± 0	3 ± 0	3 ± 0	3 ± 0	3 ± 0.49	3 ± 0.49
Thrombocytes (10^9^/L)	331 ± 116	280 ± 111	219 ± 102	161 ± 72 *	393 ± 144	270 ± 63	219 ± 76 *	112 ± 55 *
Isoprostane (ng/L)	64 ± 50	65 ± 44	60 ± 27	59 ± 23	110 ± 18	59 ± 15	89 ± 50	86 ± 50
TBARs (µmol/L)	0.23 ± 0.1	0.16 ± 0.1	0.16 ± 0.1	0.17 ± 0.03	0.20 ± 0.04	0.14 ± 0.04	0.20 ± 0.1	0.20 ± 0.1
NOx (µmol/g prot.)	1.1 ± 0.6	1.2 ± 0.5	2.2 ± 0.5 *	1.8 ± 0.7	0.8 ± 0.5	1.5 ± 1.1	1.8 ± 0.9 *	1.8 ± 0.9 *

## Data Availability

The data presented in this study are available on request from the corresponding author.
